# Electric Power Self-Supply Module for WSN Sensor Node Based on MEMS Vibration Energy Harvester

**DOI:** 10.3390/mi9040161

**Published:** 2018-04-01

**Authors:** Wenyang Zhang, Ying Dong, Yushan Tan, Min Zhang, Xiang Qian, Xiaohao Wang

**Affiliations:** 1 Graduate School at Shenzhen, Tsinghua University, University Town of Shenzhen, Shenzhen 518055, China; wenyang-16@mails.tsinghua.edu.cn (W.Z.); tanyushan92@126.com (Y.T.); zhang.min@sz.tsinghua.edu.cn (M.Z.); qian.xiang@sz.tsinghua.edu.cn (X.Q.); wang.xiaohao@sz.tsinghua.edu.cn (X.W.); 2 Tsinghua-Berkeley Shenzhen Institute, University Town of Shenzhen, Shenzhen 518055, China

**Keywords:** wireless sensor network, vibration energy harvesting, MEMS, electromagnetic conversion

## Abstract

This paper proposes an electric power self-supply module for the wireless sensor network (WSN) sensor node. The module includes an electromagnetic vibration energy harvester based on micro-electro-mechanical system (MEMS) technology and a processing circuit. The vibration energy harvester presented in this paper is fabricated by an integrated microfabrication process and consists of four similar and relatively independent beam vibration elements. The main functions of the processing circuit are to convert the output of the harvester from unstable alternating current (AC) to stable direct current (DC), charge the super capacitor, and ensure the stable output of the super capacitor. The preliminary test results of the harvester chip show that the chip can output discontinuous pulse voltage, and the range of the voltage value is from tens to hundreds of millivolts in the vibration frequency range of 10–90 Hz. The maximum value that can be reached is 563 mV (at the vibration frequency of 18 Hz). The results of the test show that the harvester can output a relatively high voltage, which can meet the general electric power demand of a WSN sensor node.

## 1. Introduction

As the core technology of the Internet of Things (IoT), the wireless sensor network (WSN) has become a topic of lively debate in recent years, and the scope of applications involving a WSN is rapidly expanding to include equipment monitoring, environmental information acquisition, detecting the link of entities, etc.

The WSN consists of a large number of sensor nodes; thus, the energy supply for the system is an important issue. Because battery life is limited, and it is hard to replace the batteries of a large quantity of sensor nodes regularly, the technology for harvesting energy from the environment around the sensor nodes has important significance. With the wide application of micro-electro-mechanical system (MEMS) sensors in WSN nodes, the general energy consumption of the WSN sensor node has been reduced to microwatts. Using an energy harvester with a power management strategy to achieve electric power self-supply is considered to be an effective way to solve the problem of the WSN node power supply. Considering that vibration energy widely exists around WSN sensor nodes, such as bridges, buildings, and vehicles, it is rational to harvest vibration energy for the power supply.

An electromagnetic energy harvester is more efficient in low frequency [1]. The main vibration module of electromagnetic energy is beams or cantilever beams. Thus, dynamic studies about microbeams [2,3,4,5,6] have been published to improve the theoretical model. Furthermore, initial deformation [7,8,9,10,11,12], damping [13,14], and complex structures [15,16] are important influences on the resonant frequency of the microbeams, because manufacturing errors in MEMS processes are unavoidable. The cantilever vibrates with greater amplitude and may introduce more nonlinear calculations [17,18,19]. The vibration energy harvester is more inclined to use the microbeam structure. Kinetic studies of microplates [20,21,22,23,24,25] provide guidance for the movement of trays commonly found in such harvesters. Improving energy conversion efficiency and developing MEMS compatible processes are the main goals of the electromagnetic energy harvesters. Shearwood and Yates et al. designed and produced the electromagnetic micro-vibration energy harvester at the millimeter scale in 1997 [26]. Since then, electromagnetic vibration energy harvesters have basically followed their structural characteristics. Beeby et al. designed and fabricated an electromagnetic induction energy harvester based on a cantilever structure in 2007 [27]. The cantilever of this harvester was fixed with bolts and nuts, so its size was relatively big and needed more steps to assemble. However, the cantilever structure is a common vibration unit and attracts much attention. The rated output voltage of the harvester is 428 mV. Beams with fixed ends are also common structures in vibration harvesters [28]. In addition, the fixed beam can work at both fundamental and high-order resonant frequencies, which means it can work at a wider frequency band; this is a useful method for broadening the bandwidth of vibration energy harvester. The harvester can generate power of 0.6 μW and 3.2 μW at the first and second resonant frequencies. Compared with bonded magnets [29], electroplated magnets [30] reduced the size of the device and greatly improved compatibility with MEMS. Although the stability and magnetic energy of the electroplated permanent magnet are weaker than that of the bonded magnet, integrating a magnet on the substrate is still the general trend in electromagnet vibration harvesters in order to make an integrated chip. Simultaneously, the multimodal vibration energy harvester represents another research direction. A MEMS harvester with nine resonant peaks from 189 Hz to 662 Hz was fabricated in 2015 [31]. The open-circuit output voltage can reach 0.13 mV maximally. Generally, high-power harvesters should have greater volume and complex processing, while broadening bandwidth would lead to low-output power.

The output voltage and power of piezoelectric [32,33,34] and capacitive [35,36] vibration energy harvesters are generally very low at the microscale.

In this paper, we design, fabricate, and test a new electromagnetic vibration energy harvester based on MEMS technology. An energy management circuit frame is also proposed to complete an electric power self-supply module based on the MEMS energy harvester. The harvester in this paper shows better output performance and it shows the potential for low-power system applications.

## 2. Materials and Methods

### 2.1. The Theoretical Model of the Harvester

The basic physical principle of the micro-electromagnetic vibration energy harvester is Faraday’s law of electromagnetic induction. When the magnetic flux through a closed-circuit changes, the induced current will occur in the circuit. In an electromagnetic vibration harvester, a movable magnet with an immovable coil is a rational adopted form. Environmental vibration forces the magnet of the harvester to move in the magnetic field, and the magnetic flux changes as well. Thus, an induced current occurs in the coils, and mechanical energy is transferred to electric energy.

In 1996, Yates et al., conducted a theoretical analysis of energy conversion based on the second-order vibration model [37]. Since then, many articles have also applied a similar model for theoretical analysis [38,39]. At present, the theory of the electromagnetic vibration energy harvester has been relatively matured. The second-order vibration model is carried out to modeling, calculation, and qualitative analysis to provide the basis and theoretical guidance for the design of the harvester in order to effectively enhance the harvester’s output performance.

When the system is at the resonance frequency (*ω* = *ω_n_*), through calculation and derivation, there are equations to calculate the total power *P* of the second-order model of the vibration energy harvester and the power on the load *P_L_* [40]:

(1) $$P=\frac{{\left(NBl\right)}^{2}Y^{2}{\omega_{n}}^{2}}{8\zeta^{2}\left(R_{L}+R_{C}\right)}$$

(2) $$P_{L}=\frac{m{Z_{\max}}^{2}{\omega_{n}}^{3}}{16\zeta_{m}}(\frac{R_{L}}{R_{L}+R_{C}})$$

In the equations, *N* is the turns of the coils, *B* is the average flux density in the harvester, *Nl* is the total effective length of the coil, *ω_n_* is the natural frequency of the system, and *Y* is the amplitude of the excitation signal. *ζ* = *ζ_e_*+ *ζ_m_*, *ζ* is the damping ratio of the system, including the electromagnetic damping ratio *ζ_e_* and the mechanical damping ratio *ζ_m_*. Electromagnetic damping refers to the phenomenon that occurs when the conductor moves in the magnetic field: the induced current will make the conductor suffer ampere force and the direction of the ampere force will always hinder the movement of the conductor. This electromagnetic damping phenomenon stems from the principle of electromagnetic induction. The electromagnetic damping ratio is used to characterize the resistance the conductor suffered. The use of electromagnetic damping has been studied [41]. *R_L_* is the resistance of the load, *R_C_* is the resistance of the coil, *m* is the mass of the moving section, and *Z* is the displacement of the moving section.

According to the two equations, a MEMS electromagnetic vibration energy harvester should follow several methods during design and fabrication to improve its output performance: increasing the mass of the magnet, the effective length of the coil, and the magnetic flux density.

### 2.2. Design and Fabrication of the Harvester

**Figure 1:**
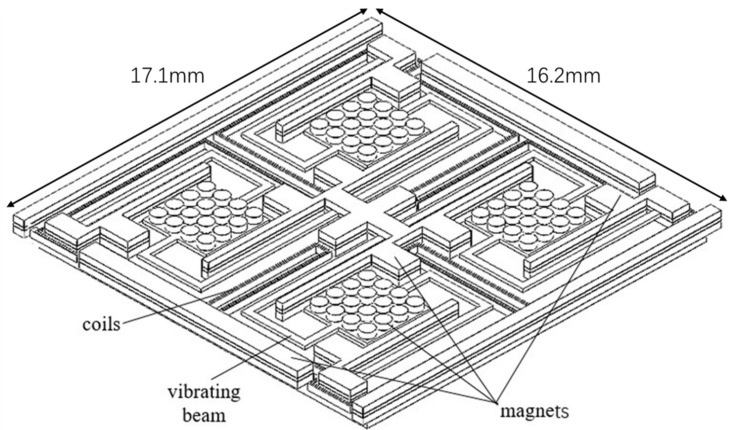
Diagram of the vibration energy harvester.

Considering the design rules discussed in the previous section, a new vibration energy harvester (Figure 1) has been designed and fabricated. The harvester consists of four similar and relatively independent beam vibration elements. A square tray with sixteen circular magnets is arranged in the middle of each beam. Other immovable magnets are plated in the middle and the edge of the harvester. Copper coils mounted between the beams and immovable magnets. Considering the structure of the beam, the simulation results of Awaja et al. [42] show that a beam with more bends and longer length has a greater displacement of the magnets and a lower resonant frequency. The four beams were designed to have different lengths to harvest vibration energy in different frequencies. The use of a curved beam can make better use of space and help to reduce the size of the harvester.

For the selection of the vibration beams material [43], the main material parameter to be considered is the elastic modulus. The common electroplating material, copper, has a suitable Young’s modulus, so it can achieve greater vibration displacement and better reliability, and copper is easy to use as electroplating in manufacturing because of its cheaper price. Therefore, copper has been chosen as the material of the vibration beams.

One of the major problems of the vibration energy harvester with MEMS processes and electroplated permanent magnets is that the output voltage is very low. The design in this work consists of four vibration beams to increase its output. First, four vibration modules are integrated into a harvester whose output is higher than that with only one beam. Second, in our design, the lengths of the four beams are different so that the resonance frequency, and therefore the bandwidth of the harvester is broadened and the harvester can output voltage in a wider frequency range. In addition, the immovable permanent magnets filling in the space of the harvester can supply a stronger magnetic field, following the rules to improve the output mentioned in the previous section.

**Figure 2:**
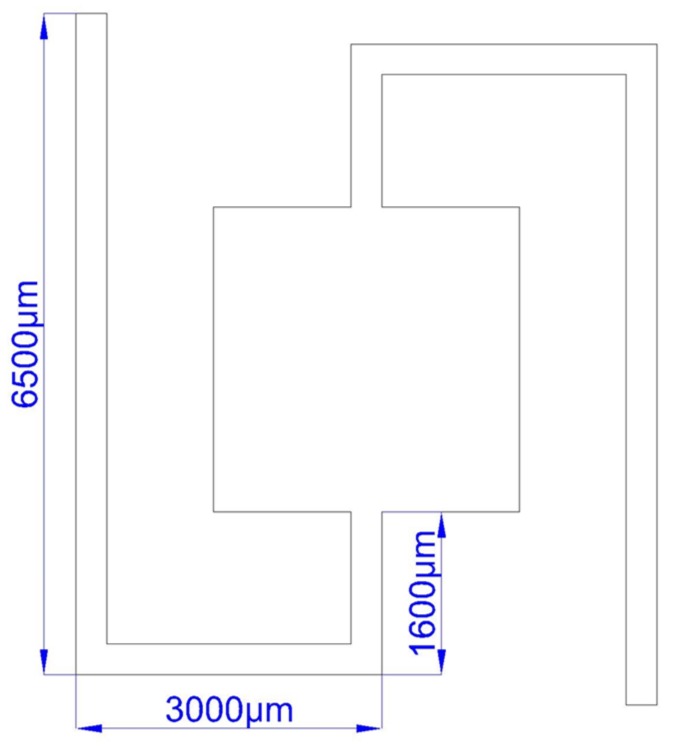
Length of the beam.

Because both ends of the beams are fixed while the trays are impending, when forced by external vibration, the trays, and the magnets on the trays, will vibrate. The magnetic flux in the coils changes with the vibration of the permanent magnets on the trays, which results in an induced current in the coils. The area of the harvester is 17.1 mm × 16.2 mm. The total lengths of the single beams are adjusted to 10,500 μm, 10,800 μm, 10,820 μm, and 11,100 μm, respectively, as shown in Figure 2, and the width of the beams is 300 μm. At this point, their resonant frequency can be simulated as 65.5 Hz, 67 Hz, 68.5 Hz, and 70 Hz. With other parameters unchanged, the longer the length of the beam, the lower the resonant frequency.

**Figure 3:**
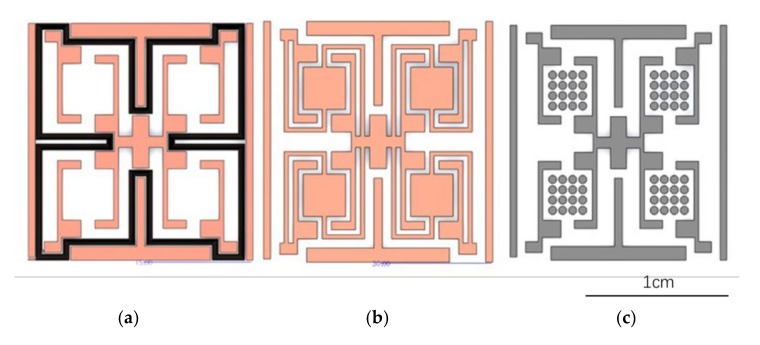
The three masks used in the fabrication process.

The fabrication processes of the harvester are as follows:

(1) The main purpose of the first step is to manufacture copper coils. First, 500 nm silicon dioxide is deposited on a 4-inch wafer as an insulation layer, and 50 nm Ti and 200 nm Cu are sputtered as a seed layer. Next, a 30 μm negative photoresist is deposited, and then, mask 1 is used (Figure 3a) to cause exposure and development. The actual thickness of the photoresist is measured as an average of 35.05 μm.
(2) The result of the second step is beams and trays. Accordingly, 30 μm Cu is plated and 200 nm Cu are sputtered as a seed layer. Next, a 15 μm negative photoresist is deposited, and then, mask 2 (Figure 3b) is used to cause exposure and development. The actual thickness of photoresist is measured as an average of 24.98 μm.
(3) Finally, 15 μm Cu is plated. Next, a 30 μm negative photoresist is deposited, and then, mask 3 (Figure 3c) is used to cause exposure and development. Then, a 30 μm CoNiMnP permanent magnet is electroplated. Finally, all photoresist and seed layers are etched. The actual thickness of photoresist is measured as an average of 33.60 μm. The average thickness of CoNiMnP is 18 μm.

Wet etching was used to remove the photoresist, and dry etching was used to remove the seed layer. Acetone with a water bath at 60 °C was used to remove any residual photoresist.

In order to remove the photoresist expediently, KMPR negative photoresist (MicroChem, Westborough, MA, USA) was chosen for the lithography process. The production results and details are shown in Figure 6. The fabrication process and the results of the harvester basically meet the abovementioned design requirements and any errors of dimensional accuracy are within the acceptable range.

Three masks were used during the fabrication process, as shown Figure 3. The black, orange, and gray parts in Figure 3 indicate the position of the coils, plated copper, and magnets, respectively. The general choice for plating permanent magnets is CoNiMnP. Sun et al. [44] studied the CoNiMnP permanent magnet plating process, using a thick magnet array to replace a magnet film and achieving higher performance. In addition, they gave the ratio of the electrolytes in the electroplating process. As for the shape of the magnets, cylinder arrays magnets show better retentivity and energy density than cubic arrays. For cylinder arrays, the retentivity and energy density increase as the radius decreases.

### 2.3. Design of the Circuit Frame

An energy harvester is the first part of an electric power self-supply module. Next, an electric energy management circuit is needed to control the energy conversion and store process. The circuit in solar- [45,46], thermal- [47], and wind-based [48] energy harvesting can be examples for a vibration energy harvester.

**Figure 4:**
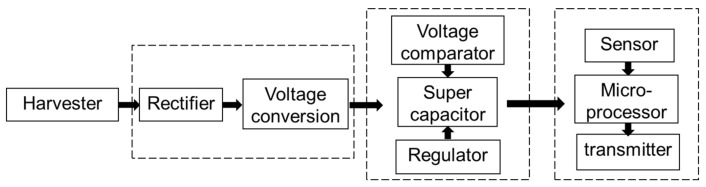
The framework of the processing circuit.

The vibration energy collected by the harvester is converted to electric power, but the power cannot be stored directly by a capacitor or battery. A subsequent circuit is needed to do an AC/DC conversion, charge the super capacitor and battery, and control the capacitor to output a stable voltage for the other chips in a WSN node. The circuit includes a rectifier, a voltage conversion module, a super capacitor, a voltage comparator, and a regulator, as shown in Figure 4. Similar circuits have been used for solar, thermal [49], and triboelectric energy [50].

**Figure 5:**
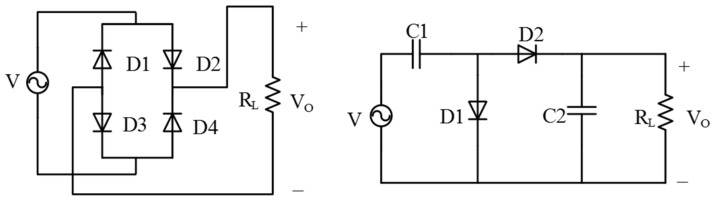
The bridge rectifier and the boost rectifier.

The general rectifiers are a full bridge circuit and boost rectifier. When input is the same (100 mV), the latter can output a higher but fluctuating signal, as shown in Figure 5. In simulation, when the input voltage is low, the second circuit can output a higher voltage than the full bridge circuit because the capacitor can store electricity power. The diodes and capacitances used in the simulation are 1BH62 and 10 μF, respectively.

A super capacitor is chosen as the energy storage component, because a super capacitor shows better power density and cycle life than a battery, as shown in Table 1. In a natural environment, frequent charge and discharge is necessary. The energy storage does not need to supply energy for the system continuously, because the transmitter receives and transmits signals periodically. More cycle times would ensure that the WSN has a longer life. Though the energy density is smaller than that of a battery, the energy stored in a super capacitor is enough to drive a WSN node.

The voltage conversion module is important in the whole circuit, because it is responsible for charging electricity into the capacitor. There are different kinds of chips for different natural energies. They usually have a unique requirement for the input voltage. For instance, solar energy can achieve volts level while vibration energy is at millivolt or lower.

The comparator can detect the voltage of the super capacitor and control the charge and discharge status of the system. The output voltage of the capacitor will decrease with the discharge and the voltage change may affect the other parts in the sensor node. The regulator is used to reduce the output voltage fluctuations of the capacitor.

## 3. Results

**Figure 6:**
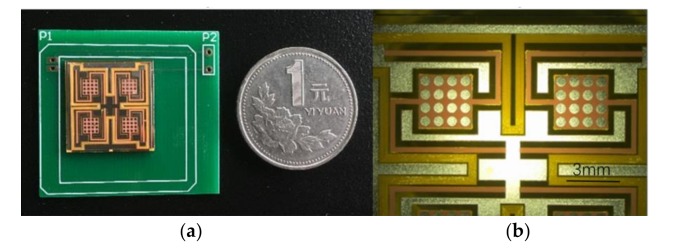
(**a**) Comparsion between the harvester and a coin, (**b**) The details of the harvester.

The harvester is fixed on printed circuit board (PCB), and the leads are connected, as shown in Figure 6. The equipment required for testing include, a vibration table, an accelerometer, a waveform generator, an oscilloscope, and a power amplifier. The waveform generator outputs a sine wave to power the amplifier, then the power amplifier drives the vibration table to vibrate and forces the harvester settled on the vibration table to vibrate. The output from the harvester is detected by the oscilloscope. An accelerometer is fixed with the harvester to detect the acceleration of the harvester. The two pins of the coils in the harvester are connected with the pins of P1 by a gold wire welding machine. P1 and P2 are connected by an internal wire of the PCB to reduce error of signal, and P2 is used to weld with the consecutive circuit wire.

**Figure 7:**
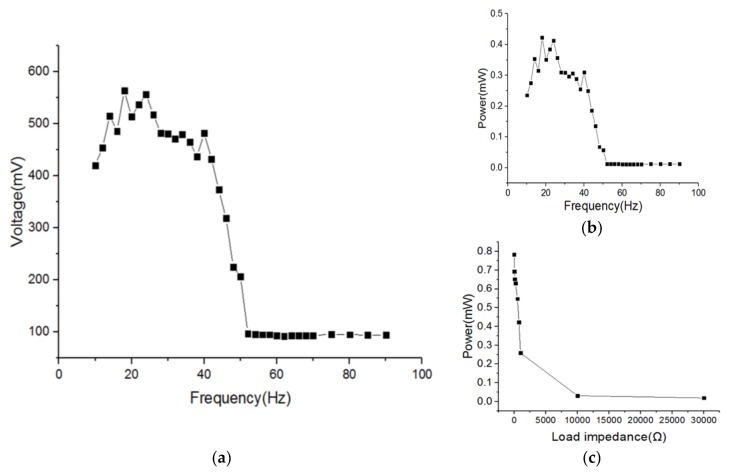
(**a**) Output voltage versus frequency, (**b**) output power versus frequency, and (**c**) output power versus load impedance.

As shown in Figure 7, when the acceleration is of 1g and the load resister is 750 Ω, the test results of the harvester show that the harvester chip can output discontinuous pulse voltage, and the range of the voltage value is from tens to hundreds of millivolts in the vibration frequency range of 10–90 Hz. The maximum value that can be reached is 563 mV (at the vibration frequency of 18 Hz) and power is 0.423 mW. The harvester shows better performance in the low frequency range (10–40 Hz), the output voltage is above 200 mV, and power is above 0.2 mW. The internal resistance of the harvester is very small (about 1 Ω) compared with the load. When the load resistance is close to the internal resistance of the harvester, the output power is high, and the maximum power reaches 0.784 mW as the load is 1 Ω, and as the external load gradually increases, the output power becomes lower. When the load is larger than 10,000 Ω, the output power is lower than 0.1 mW.

Compared with the simulation result, the resonant frequency is lower. The possible reasons are the residue of the photoresist and the residual stress and effect of electricity on the movement of microbeam [51]. It is possible that the tray of magnets may undergo rotation if it is not strictly perpendicular to the direction of the vibration, and the efficiency of the device may decrease due to the rotation. Therefore, proper installation is required.

Table 2 shows a comparison of the output performance of different harvesters of similar sizes. The harvester in this paper shows better output performance than other harvesters. It shows the potential for low-power system applications.

## 4. Discussion

In experiments, the harvester can output discontinuous pulse voltage under sinusoidal vibration signals. The pulse voltage can reach more than 400 mV in the range of 10–40 Hz, and the maximal value is about 563 mV at 18 Hz. Therefore, the goal to increase the output of the harvester has been achieved. In structure simulation, the resonant frequency of the four beams are 65.5 Hz, 67 Hz, 68.5 Hz, and 70 Hz respectively. Compared with the simulation, the resonant frequencies are lower, and the high-output frequency band is wider. Any remaining residue from the photoresist removal stage, deformation caused by stress, and manufacturing errors may be the reasons for these phenomena. Moreover, the output of the four trays may have phase differences when vibrating, which may cause a decrease in the output voltage. In addition, the harvester cannot output a complete sinusoidal signal, but can only output separate voltage pulses, which indicates that the vibration modules of the harvester may not achieve ideal forced vibration.

A simple comparison of rectifiers is given in this paper. It can become a reference for choosing a proper rectifier for the circuit. The framework of the processing circuit is a guiding ideology of circuit design for a WSN node.

## 5. Conclusions

In this paper, a new electromagnetic vibration energy harvester based on MEMS technology has been designed, fabricated, and tested. An energy management circuit frame was also proposed to complete an electric power self-supply module based on the MEMS energy harvesters. The harvester was fabricated by photolithographic and electroplated processes. The harvester contains four vibration beam structures, a multi-turn coil surrounding the vibration structures, and electroplated CoNiMnP as the magnetic material. The main concerns in the design of the harvester were increasing bandwidth and integrating with the MEMS manufacturing process. The results show that under a sinusoidal signal excitation, the harvester can output discontinuous pulse voltage, the maximal value of which is 563 mV at the vibration frequency of 18 Hz. The function of the energy management circuit is to convert the output of the harvester from unstable AC to a stable DC, charge the super capacitor, and ensure stable output of the super capacitor. The output of the harvester is high enough to support the subsequence circuit, and the framework of the circuit is completed for an electric power self-supply module for a WSN sensor node. The power consumption of the commercial products is tens of milliwatts, and the output power of the harvester is 0.423 mW; thus, it can operate intermittently. It takes several minutes to charge the super capacitor.

## Figures and Tables

**Table 1:** Comparison of super capacitor and lithium ion battery.

Energy Storage	Energy Density (Wh/kg)	Power Density (W/kg)	Cycle Life
Super Capacitor	0.2–20	7000–18,000	>100,000
Lithium Ion Battery	100–150	100–150	<1000

**Table 2:** Comparison of output performance of different harvesters.

Energy Storage	Voltage	Power
This Paper	563 mV	0.423 mW
Yang, B. [4]	-	3.2 μW
Liu, H. [7]	0.13 mV	16.012 × 10^−6^ μW
Wang, P. [29]	18 mV	0.61 μW
Özge Zorlu [52]	9.5 mV	363 nW
